# Factors affecting women's disclosure of domestic abuse and healthcare professionals’ preparedness to identify and respond: a systematic literature review

**DOI:** 10.3389/fgwh.2026.1702307

**Published:** 2026-10-07

**Authors:** Mandu Stephen Ekpenyong, Khatidja Chantler, Gillian Janes

**Affiliations:** 1 School of Nursing and Public Health, Manchester Metropolitan University, Manchester, United Kingdom; 2 School of Nursing, Anglia Ruskin University, Chelmsford, United Kingdom

**Keywords:** domestic abuse, healthcare professionals, preparedness, readiness, systematic literature review

## Abstract

**Purpose:**

Domestic abuse (DA) is a significant public health and human rights issue with profound physical, psychological, and social consequences. Healthcare settings provide important opportunities for identification and support; however, disclosure by women remains inconsistent, and healthcare professionals’ readiness to identify and respond varies widely. This qualitative systematic review synthesised evidence examining factors influencing women's disclosure of DA within healthcare contexts and healthcare professionals’ preparedness to respond.

**Methods:**

A systematic search of MEDLINE, Embase, CINAHL, PsycINFO, and Scopus was conducted for studies published between 2000 and 2023. Additional studies were identified through reference searching. The review followed PRISMA guidance and was informed by the SPIDER framework. Qualitative and mixed-methods studies with extractable qualitative findings were included. Methodological quality was appraised using the Critical Appraisal Skills Programme checklist. Findings were synthesised narratively and interpreted using a socio-ecological model to capture multilevel influences on disclosure and response.

**Results:**

Disclosure emerged as a relational and socially mediated process shaped by trust, perceived safety, validation, confidentiality, and continuity of care. Forms of “borrowed trust,” mediated through social networks, influenced women's willingness to engage. Healthcare professionals’ preparedness was determined by competence, confidence, emotional readiness, clarity of safeguarding responsibilities, and access to referral pathways. Structural factors, including limited consultation time, workload pressures, and organisational ambiguity, constrained effective response. Importantly, disclosure alone did not ensure improved outcomes where system readiness was inadequate.

**Conclusion:**

Improving responses to DA requires multilevel strategies extending beyond individual training. Sustainable progress depends on organisational leadership, protected time, clear referral systems, and alignment across relational and structural domains to support safe disclosure and meaningful intervention.

## Introduction

Domestic abuse (DA), also referred to as domestic violence or intimate partner violence (IPV), encompasses a range of behaviours occurring within intimate or familial relationships. In England and Wales, the Domestic Abuse Act 2021 provides a statutory definition covering physical, sexual, emotional, psychological, controlling or coercive, threatening, and economic abuse between individuals aged 16 years or over who are personally connected (1). This legislative definition aligns with the broader United Nations framing of violence against women as any act of gender-based violence resulting in, or likely to result in, physical, sexual, or psychological harm or suffering, including threats, coercion, or deprivation of liberty, whether occurring in public or private life (2). These definitions situate domestic abuse as both a criminal justice issue and a violation of women's fundamental human rights.

Domestic abuse is also a serious public health issue and a violation of women's human rights (3). Globally, the World Health Organization estimates that approximately one in three women aged 15 years and over have experienced physical and/or sexual intimate partner violence or non-partner sexual violence in their lifetime (4). In England and Wales, 5.0% of adults aged 16 years and over reported experiencing domestic abuse in the year ending March 2022, equating to approximately 2.4 million adults (5). In the United States, lifetime estimates suggest that one in four women have experienced sexual violence, physical violence, or stalking by an intimate partner (6). An estimated 1 in 4 women and 1 in 10 men have experienced sexual violence, physical violence, and/or stalking by an intimate partner during their lifetime, with IPV-related impacts such as being concerned for their safety, injury, or needing victim services (6). Distinguishing between lifetime and annual prevalence is important, as annual figures capture current burden on services, while lifetime estimates reflect cumulative exposure and long-term health impact.

DA affects people of all socio-economic backgrounds, ages, educational levels, and gender identities, even though it is reported to disproportionately affect women and children (7).

Women who experience DA are reported to have poorer physical, sexual, and mental health than non-abused women. Some of the physical impacts of DA on women include injuries such as bruises, broken bones, and lost teeth (8). The sexual effects of DA on women include miscarriage and other complications of pregnancy (7, 8). Effects on mental health include depression, self-harm and suicide attempts, poor self-esteem, and stress and anxiety disorders (9). The effects of violence are more severe when women experience more than one type of abuse (physical, emotional, and coercive control) and/or multiple incidents over time (10).

The National Health Service (NHS) in the UK plays a direct role in treating victims and survivors of DA in several ways. This could include the provision of mental health services, commissioning of sexual assault referral centres, or by treating physical injuries. Healthcare Practitioners (HCPs) are therefore likely to come into contact with victims of DA as part of their routine practice. This study, therefore, set out to explore the evidence regarding healthcare professionals’(HCPs’) preparedness to identify and respond to DA following disclosure.

## Aim and objectives

The aim of this study was to synthesise evidence from empirical studies on how HCPs respond to DA among women as part of their everyday role. The objectives of the systematic review were as follows:

1. Explore women's experiences of disclosing domestic abuse within healthcare settings.
2. Examine healthcare professionals’ perceptions of their preparedness to identify and respond to domestic abuse in routine clinical practice.
3. Identify individual, interpersonal, institutional, and system-level barriers and facilitators influencing disclosure and response, using a socio-ecological framework.
4. Synthesise implications for healthcare practice, professional education, and organisational support to strengthen responses to domestic abuse.

## Definitions of key terminology used in this manuscript

1. Disclosure refers to the intentional sharing of domestic abuse experiences with a healthcare professional, whether directly or indirectly.
2. Preparedness refers to healthcare professionals’ perceived readiness, confidence, and competence to identify and respond, including knowledge of safeguarding and referral pathways.
3. Identification refers to recognising indicators of abuse through clinical interaction and assessment.
4. Response refers to actions following disclosure or suspicion, including validation, documentation, risk assessment, safeguarding, and referral.
5. Trust refers to women's expectation that healthcare professionals will act competently, confidentially, and in their best interests, including safeguarding responsibilities.

## Methods

This review was conducted and reported in accordance with the Preferred Reporting Items for Systematic Reviews and Meta-Analyses (PRISMA 2020) guidance (11) and is illustrated in Figure 1.

**Figure 1:**
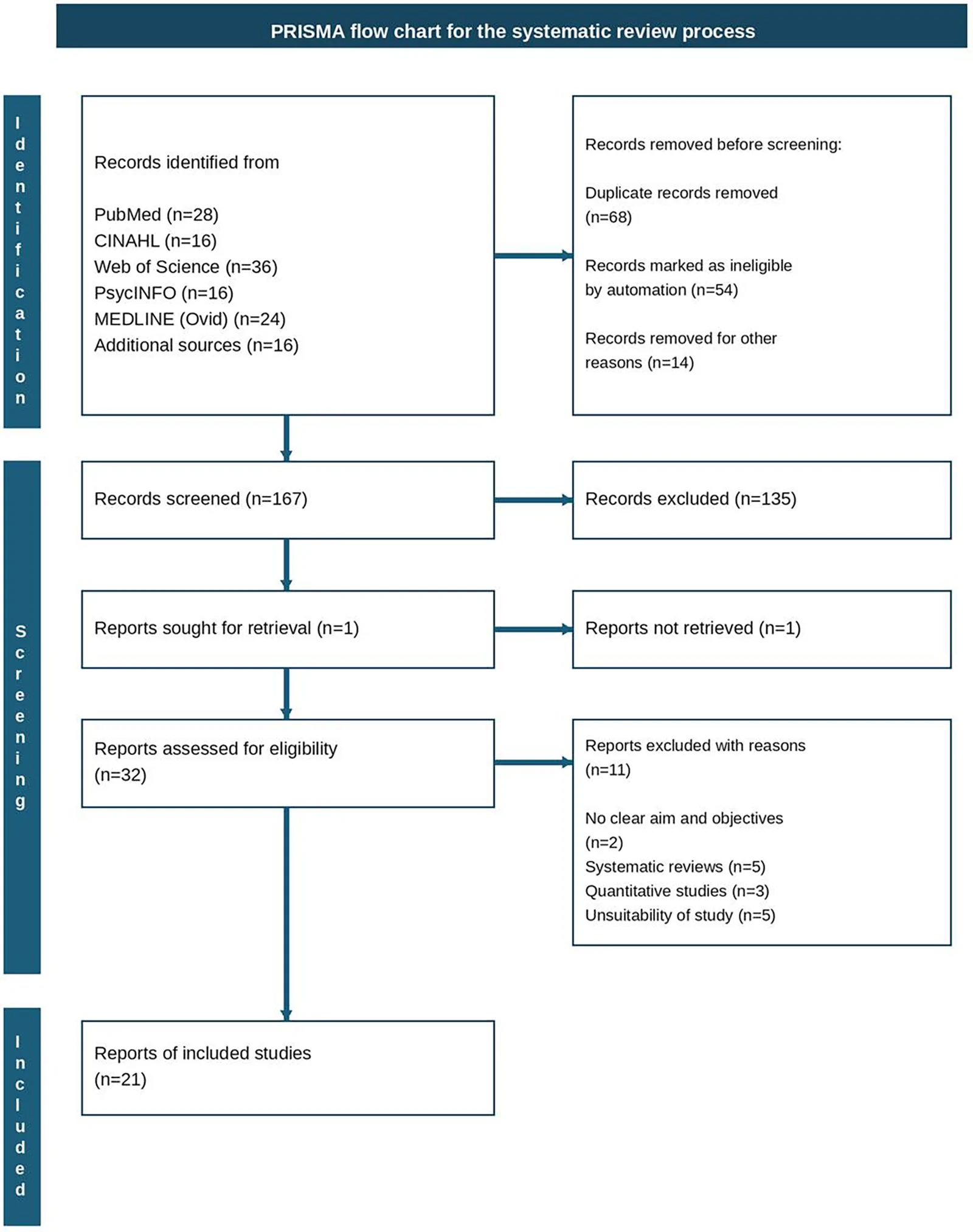
PRISMA flow chart for the systematic review process.

### Search strategy

A comprehensive, structured search of MEDLINE, Embase, CINAHL, PsycINFO, and Scopus online databases was conducted. The original search was conducted between 8 and 20 November 2022 for studies published from 2000 to 2022. In response to peer review, the search was updated to include studies published up to 2023, resulting in the inclusion of two additional eligible studies. We also searched Google Scholar and references from included articles for additional studies. The review team iteratively developed the search string and tested it before use.

The review applied the SPIDER framework—Sample, Phenomenon of Interest, Design, Evaluation, and Research type—to define the eligibility criteria and guide the selection of relevant studies. Table 1 presents how each SPIDER component was operationalised in this review.

**Table 1:** Spider framework.

SPIDER component	Application in this review
S (Sample)	Women with lived experience of domestic abuse and healthcare professionals (e.g., nurses, doctors, midwives, mental health practitioners) working in healthcare settings
PI (Phenomenon of Interest)	Women's disclosure of domestic abuse and healthcare professionals’ identification of and responses to domestic abuse
D (Design)	Qualitative study designs, including semi-structured interviews, in-depth interviews, ethnography, and focus groups
E (Evaluation)	Experiences, perceptions, attitudes, confidence, readiness, preparedness, barriers, and facilitators related to disclosure and response
R (Research Type)	Qualitative research

### Inclusion and exclusion criteria for the included studies in the review

The inclusion and exclusion of retrieved articles were determined using the following criteria:

#### Inclusion

In this review, healthcare professionals refer to regulated clinical practitioners (e.g., doctors, nurses, midwives, and allied health professionals) working in healthcare settings. Studies involving non-clinical staff (e.g., counsellors or advocates) were included only where their role was embedded within a healthcare service and findings directly related to identification, disclosure, safeguarding, referral, or clinical response.

1. Empirical/primary research using qualitative studies exploring women's disclosure of domestic abuse in healthcare settings, and/or healthcare professionals’ preparedness, readiness, or responses to domestic abuse
2. Mixed-methods studies with extractable qualitative findings
3. Published between the years 2000 and 2023
4. Published in the English language
5. Conducted in healthcare settings within high-income countries (e.g., Australia, Canada, Europe, the UK, and the USA).

#### Exclusion

Exclusion criteria were as follows: quantitative studies, mixed-methods studies where qualitative findings could not be clearly isolated, intervention studies evaluating effectiveness rather than experiences or perceptions, prevalence studies, grey literature (e.g., reports, theses, conference abstracts), and non-peer-reviewed publications.

### Search strategy (combined terms using Boolean operators)

The final keywords used in the search are presented in the following.

Population (Healthcare Professionals):

“healthcare professional*” OR “health care provider*” OR physician* OR doctor* OR nurse* OR midwi* OR “mental health professional*”

Exposure (Domestic Abuse/Violence):

“domestic abuse” OR “domestic violence” OR “intimate partner violence” OR “spousal abuse” OR “gender-based violence”

Concepts (Readiness/Response):

recogn* OR identif* OR screen* OR respond* OR readine* OR competenc* OR prepar* OR “clinical practice”

Combined search string (example):

(“domestic abuse” OR “domestic violence” OR “intimate partner violence” OR “spousal abuse” OR “gender-based violence”) AND (recogn* OR identif* OR screen* OR respond* OR readine* OR competenc* OR prepar* OR “clinical practice”) AND (“healthcare professional*” OR “health care provider*” OR physician* OR doctor* OR nurse* OR midwi* OR “mental health professional*”)

### Screening and selection of studies and quality assessment

Quality assessment was conducted for all included papers and used to describe reporting quality rather than to determine eligibility. The Critical Appraisal Skills Programme (CASP) tool for appraising qualitative studies (12) was used to assess the quality of the qualitative articles. Titles and abstracts were screened against the inclusion and exclusion criteria. Articles meeting these criteria were selected for full-text screening.

### Study selection, data abstraction, and analysis

Study selection was conducted in three stages: title screening, abstract screening, and full-text review. One reviewer (ME) screened all titles and abstracts. To enhance methodological rigour and reduce selection bias, a second reviewer (GJ) independently screened a pre-specified 25% random sample of full-text articles. This proportion was determined *a priori* in line with accepted qualitative systematic review practice, where dual full screening of all papers is not feasible but independent verification is required to enhance credibility. Discrepancies were resolved through discussion among the review team (ME, GJ, and KC) until consensus was reached. Data abstraction was conducted using a structured extraction template and independently cross-checked by two additional reviewers (GJ and KC) to ensure accuracy, completeness, and consistency. This process strengthened the dependability and confirmability of the synthesis.

The extracted data included citation, study aim(s), study population, sample size/sampling technique, study design, data analysis method, and study outcomes (see Table 2). Narrative analysis of included studies was completed according to research design, country, participant group, and type of abuse/victim. Narrative synthesis is useful for analysing diverse datasets (13). Study information was systematically tabulated, and patterns within and between studies were explored to identify commonalities, differences, and potential explanations for these.

**Table 2:** Data abstraction table (DA).

S/n	Reference	Research aim and setting	Design/Participants and recruitment strategy	Data collection and analysis strategy	Key findings
1.	Battaglia et al. (20) Country: USA	To identify characteristics that facilitate trust in the patient–provider relationship among survivors of intimate partner violence (IPV)	Twenty-seven female survivors of IPV	Semi-structured open-ended interview	Barriers: Lack of open communication hinders trust, and not respecting autonomy
		Settings: Community-based domestic violence counselling or sheltering programme in Eastern Massachusetts	Recruitment strategy: Not stated	Analysis method: Data were analysed using NU*ST, a computer software program for qualitative research.	Facilitators: Open dialog with healthcare providers was perceived as a positive trusting relationship, a sense of being heard and understood, professional competency, caring, and emotional equality.
2	Colarossi et al. (21) Country: USA	Settings: Three family planning centres in New York	Seventy-five healthcare staff participated in the focus group discussion, whilst 64 of them completed the survey.	Mixed methods using both survey and focus group discussion	Factors influencing screening for intimate partner violence include attitude, resource barriers and preparedness.
			Recruitment strategy: convenience sampling	Analysis method: Both inferential statistics and thematic analysis were conducted.	
3.	Goff et al. (22) Country: USA	To explore why healthcare professionals do not always screen patients for DA	Fifteen individuals, equally divided among physicians, dentists, and nurse practitioners	Qualitative design using individual interview	Practitioners requested specific information to enhance educational preparedness and improve clinical practice for their abused patients.
		Setting: Interviews were conducted at each participant's workplace.	Recruitment strategy: Identified through purchased membership lists of the Texas State Board of Medical Examiners, Texas State Board of Dental Examiners, and the American Academy of Nurse Practitioners	Analysis method: Not stated	
4	Hegarty and Taft (23) Country: Australia	To determine the barriers to and rates of disclosure of partner abuse by women	Twenty abused women	Survey and interviews	Barriers to disclosure: Internal barriers include thinking that abuse was not serious enough, feelings of shame or embarrassment, abuse being only emotional. External factors include doctor unable to help, being unable to talk to a male doctor, doctor not being interested in hearing about this sort of problem, and feeling intimidated by the doctor.
		Settings: Eight Victorian domestic violence services	Recruitment strategy: Purposive sampling	Analysis method: Multivariate analyses using SPSS; phenomenological analysis, conducted with Ethno4 software	Facilitators to disclosure: Doctor was sympathetic, thought the doctor would help, knew the doctor would not tell anyone, doctor listens to all my problems, and desperate and had to tell someone
5	Henderson (24) Country: Canada and UK	To explore how nurses make sense of the interface between themselves, their working environments, and their nursing actions with abused women	Forty-nine (25 Canadian nurses and 24 UK nurses) nurses raised in these countries	Individual interviews	Environment (appropriate time or place to tackle the abuse), emotional response, and professional preparation were factors reported to play a major role in how nurses respond to abuse.
		Settings: Two teaching hospitals in Canada and UK	Recruitment strategy: Not stated	Analysis method: A thematic analysis	
6	Henriksen et al. (25) Country: Norway	To gain an in-depth understanding of midwives’ experiences with routine inquiry for intimate partner violence during the antenatal period	Eight midwives providing antenatal care	Individual semi-structured interviews	Barriers: Sensitive topic, lack of resources, women did not attend antenatal care alone, and fear of revealing were the challenges that hindered nurses from asking women about DA.
		Settings: Eight Mother and Child Health Centres in Norway	Recruitment strategy: Purposive sampling	Analysis method: Content analysis	Facilitators: Alternative approaches, engagement, sensitive communication, and positive feedback from women were reported as factors that make it easier for nurses to ask women about domestic violence and abuse.
7	Heron et al. (26) Country: UK	To explore victims’ perceptions and experiences of disclosing to healthcare services and to identify barriers and facilitators of disclosure within an ethnically diverse UK sample	Twenty-nine British (eight ethnic minority) female victims	Semi-structured interviews	Barriers to disclosure: Fear, embarrassment, partner’s physical presence, partner’s manipulation of healthcare professionals, not appropriate setting, and lack of time
		Settings: A domestic violence support organisation based in the East Midlands of England	Recruitment strategy: Purposive sampling	Analysis method: Content analysis	Facilitators: Interpersonal relationship (listening skills, trust empathy) and safety (privacy and confidentiality, and home visits)
8	Keeling and Fisher (27) Country: UK	To gain a deeper understanding of women's experiences of disclosure of domestic violence to health providers	Fifteen women living in the United Kingdom who disclosed their experiences of domestic violence when accessing healthcare	Narrative interviews	Barriers to disclosure: Dismissal of women's disclosure, partial recognition, and denial
		Settings: Two women's refuges centres within the United Kingdom	Recruitment strategy: Self-nomination	Analysis method: Thematic analysis	Facilitators to disclosure: appropriate support and advice
9	Mauri et al. (28) Country: Italy	To explore midwives’ knowledge and clinical experience of domestic violence among pregnant women, with particular emphasis on their perceptions of their professional role	Fifteen community midwives	Semi-structured interviews	Barriers to identifying DA include difficult-to-recognise domestic violence because of limited knowledge of the most common signs and symptoms of violence, a lack of training, cultural taboos, and the women's unwillingness to disclose abuse.
		Settings: Fifteen hospital and community midwives working in the local health district of Monza and Brianza in northern Italy	Recruitment strategy: Purposive sampling	Analysis method: Content analysis	Facilitators to identifying DA include healthcare professionals’ relationships with the women, specific professional training, screening tools, and active “professional involvement” in identifying and managing family violence.
10	McCauley et al. (29) Country: UK	To investigate the knowledge and perceptions of domestic violence among doctors who provide routine antenatal and postnatal care at healthcare facilities	Twenty-five doctors working in public and private hospitals and five officials involved in domestic violence policy development in Islamabad, Pakistan	Semi-structured interviews	Most doctors caring for women during and after pregnancy have a good understanding of the problem of domestic violence and have the desire to provide support for women who report domestic violence.
		Settings: Healthcare facilities	Recruitment strategy: Purposive sampling	Analysis method: Thematic analysis	Lack of resources, lack of consultation time, and lack of effective referral pathways or support were the main barriers to the provision of quality care.
11	McGarry (30) Country: UK	To explore the experiences of clinical staff in responding to disclosure of domestic violence and abuse, and to evaluate the effectiveness of training and support provided by a dedicated DA Nurse Specialist across one acute National Health Service Trust in the UK	Eleven clinical staff based in one acute care Trust in the UK	Semi-structured interviews	Factors reported to affect healthcare professionals’ role in responding to DA include the complexity of presentations of abuse, practitioner–patient relationships, and support from the DA nurse
		Settings: One NHS Hospital Trust which offers a range of in-patient, out-patient, emergency, and specialist services	Recruitment strategy: Not stated	Analysis method: Analytic Framework	
12	McGarry and Nairn (31) Country: UK	To explore the perceptions of ED staff about the perceived value and utilisation of a new DA nurse specialist role that has been created in one ED in the UK	Sixteen clinical staff	A qualitative design using semi-structured interviews	Time constraints: Taking things at face value, education and training, and professional and personal support
		Settings: In one large regional ED in the UK	Recruitment strategy: Purposive sampling	Method of analysis: Data analysis was informed by the Analytic Hierarchy Model (Ritchie and Lewis, 2003)	
13	Po-Yan et al. (32) Country: Australia	To explore how doctors perceived the concepts of readiness and preparedness to identify and respond to IPV against female patients	Nineteen doctors (eleven females and eight males) practising in primary care	Semi-structured interviews	Doctors’ readiness was influenced by self-efficacy, emotional readiness, motivational readiness, and attitudinal readiness. Factors that impact doctors’ perceived “preparedness” were IPV knowledge and communication skills.
		Settings: Primary healthcare centres	Recruitment strategy: Purposive sampling	Analysis method: Thematic analysis;	
14	Rose et al. (33) Country: UK	To explore the facilitators and barriers to disclosure of domestic violence from a service user and professional perspective	Qualitative study; 18 mental health service users and 20 mental health professionals	one-to-one interviews	Barriers: Fear that disclosure can lead to further violence, fear that health professionals may not believe their disclosure
		Settings: Deprived South London borough	Recruitment strategy: Purposive sampling	Analysis method: Thematic analysis	Facilitators: Supportive and trusting relationship between the individual and professional
15	Spangaro, et al. (34) Country: Australia	To test, among Indigenous women, a model for decisions on whether to disclose intimate partner violence in the context of antenatal routine screening	Qualitative study; 12 Indigenous women 28 + weeks pregnant attending antenatal care	Semi-structured face-to-face interviews.	Women's decisions to disclose abuse were shaped by cultural safety. Cultural safety included elements such as lack of trust, lack of empathy, and lack of good relationship between the victims and HCP.
		Settings: Five Aboriginal and Maternal Infant Health Services (two urban and three regional), and one mainstream hospital	Recruitment strategy: Purposive sampling	Analysis method: Qualitative Configurative Analysis	Direct asking of questions and a sense of safety from institutional control were reported to positively influence women's decisions to disclose abuse.
16	Sundborg et al. (35) Country: Sweden	To improve the understanding of district nurses’ experiences of encountering women exposed to IPV	Twenty district nurses	Open in-depth interviews	Barriers to asking women about abuse include a lack of strategies for asking about IPV, a lack of ideas about their professional roles and reaction, and beliefs about women exposed to IPV.
		Settings: Primary healthcare centres run by Stockholm County Council	Recruitment strategy: Random selection	Analysis method: Grounded theory approach	Facilitators: Feeling that the women had confidence in them, recognising signs of IPV, having strategies for asking about IPV, knowing how to deal with the answer, and having a supportive environment
17	Trevillion et al. (36) Country: UK	To explore the acceptability of routine enquiry and experiences of responding to domestic violence from service user and professional perspectives	Twenty-four community mental health service users and twenty-five professionals	Individual interviews	Two major themes identified: Positive experiences of help-seeking including receiving acknowledgement for the abuse and support for their multiple needs
		Setting: Mental health services team in South London borough	Recruitment strategy: Purposive sampling	Analysis method: Thematic analysis	Negative experiences include non-validating responses from clinicians following disclosure, discrimination, and an absence of support from services. Again, reporting requirements and unclear referral pathways caused difficulties in assessment and management of DA.
18	Wyatt et al. (37) Country: Australia	To identify if recently licensed associate degree-prepared registered nurses screen for intimate partner violence, how they screen, which patients are screened, and how pre-licensure education and current workplace training have influenced these screening decisions and behaviours	Sixteen nurses	Individual interviews	Preparedness, discomfort, disenchantment, presumptuousness, and evolving realisations were factors reported as impacting nurses’ ability to screen for DA.
		Settings: Specialised websites, including Facebook pages specifically for nurses and Allnurses.com.	Recruitment strategy: Purposive sampling	Analysis method: Constructivist Grounded Theory	
19	Yeung et al. (38) Country: UK	To explore the perceptions and experiences of general practitioners (GPs) and practice nurses on addressing domestic violence before and after participation in a domestic violence training and support programme	Eleven GPs and six nurses Recruitment strategy: A convenience sample of participants in a multidisciplinary domestic violence training and support programme in East London and Bristol	Semi-structured interview	Barriers to inquiry included lack of time, lack of experience, lack of awareness of community resources, and unavailability of effective interventions post-disclosure. Longstanding relationships with patients were cited both as barriers and facilitators to domestic violence disclosure.
		Setting: Twenty-four general practices participating in the intervention arm of the IRIS trial in Hackney and Bristol		Analysis Method: The framework approach	
20.	Usanov et al. (39) Country: Australia	To explore the Australian healthcare providers’ perspectives on factors influencing disclosure and screening for domestic violence	Ten community healthcare providers working with perinatal women in Greater Western Sydney, NSW, Australia	Qualitative design using in-depth semi-structured interview	Healthcare staff: Not knowing what to do: “Fear of opening a can of worms,” lacking knowledge and awareness: “Education's a huge thing,” time pressure
		Study setting: Greater Western Sydney, New South Wales, Australia	As diversity in disciplines was sought, participants included Women's Health Registered Nurses, General Practice Registered Nurses, General Practitioners, or Privately Practicing Midwives	Analysis Method: Thematic analysis guided by Braun and Clake (2006)	Women: Do not want someone to know, the waiting time, shame, fear of the ramifications
			Recruitment: Recruitment was done using the snowballing method.		
21.	Branjerdporn et al. (40) Country: Australia	To explore women's experiences of IPV screening at an Australian maternity service	A convenience sampling method was used to recruit women.	In-depth, semi-structured interviews were conducted for nine (9) pregnant women.	System consideration, communication and support, asking about IPV
		Setting: In a large tertiary hospital maternity service		Data collected were analysed thematically using NVivo 12 Pro.	

### Socio-ecological model

The socio-ecological model (SEM), drawn from the field of public health, was used to frame this review. The SEM was adopted not merely as a descriptive framework but as an analytical lens to interpret how multilevel determinants interact to shape disclosure and professional response. The SEM conceptualises behaviour as embedded within nested systems—individual, interpersonal, institutional, and societal—allowing this review to move beyond listing barriers and facilitators towards understanding their interdependence.

Using elements of the SEM, this systematic literature review explored the factors contributing to women's disclosure of abuse and healthcare professionals’ readiness to recognise, identify, and respond to abuse. The SEM suggests that we all experience more than one type of nested system, with bi-directional influences within and between systems. The SEM approach integrates different levels for taking action, including the individual, interpersonal, and social; social and health; and health systems (14). Although the components of the SEM remain the same and can be used in a range of settings, the specific examples within each component will vary depending on the population group (15). The SEM recognises that people's health behaviours are embedded within larger interactive and overlapping social systems that influence DA outcomes (16).

This model offers a broad-based conceptualisation of the complex interactions among four levels—individual, interpersonal and social, social and health system, and health factors—that serve as barriers and facilitators to women's disclosure of abuse and healthcare professionals’ readiness to respond to DA disclosure (17). The SEM acknowledges the impact of individual factors and interpersonal relations, as well as acknowledging the influence of systems on outcomes (18). It also recognises the influence of interpersonal and social factors on both women's disclosure of abuse and healthcare professionals’ readiness to respond to DA. Factors at the four levels substantially influence the increase or decrease in healthcare professionals’ readiness to respond to DA and women's disclosure of DA (19).

## Results

### Characteristics of included studies

Of the 21 included studies (20, 22, 24–40), 20 used a qualitative approach. Data collection methods included semi-structured interviews (24–26, 28–34, 36–40), narrative interviews (27), ethnographic interviewing (20), and open in-depth interviews (21, 32). The remaining study involved mixed-methods research and collected data using both survey and focus group discussion (21, 22). Eight of the studies were conducted in the UK (26, 27, 29–31, 33, 36), six were conducted in Australia (23, 32, 34, 37, 40, 41), and three in the USA (20–22). Five of the studies were conducted elsewhere: one each in Italy (28), Norway (23), Sweden (35), and one was dual-centre, spanning the UK and Canada (24). The sample sizes in most of the qualitative studies were relatively small. Sample sizes ranged from 8 (25) to 49 (24). However, this does not detract from the quality of these studies, as qualitative research does not require a large sample size, where the aim is not to generalise but to explore in depth the nature of a given phenomenon (39).

Study participants in the reviewed articles included mental health users, mental health professionals, pregnant women attending antenatal care (ANC), doctors and nurses, officials involved in domestic violence policy development, women who had experienced DA, and community midwives. Participants were recruited using varied recruitment methods. These included purposive sampling, random selection, convenience sampling, and self-nomination. Participants in the reviewed studies were recruited mostly through family planning centres, general practice, community-based violence counselling centres and shelters, refugee centres, and specialist websites. The included studies involved a range of analytic approaches, including thematic and content analysis. Among the 20 reviewed articles, the oldest was published in 2001 and the most recent in 2023. Ethical approval was reported in all the reviewed articles.

### Thematic synthesis

Narrative synthesis yielded two overarching themes reflecting the relational and structural determinants of disclosure and professional response in healthcare settings. Themes represent higher-order conceptual groupings that capture patterned meanings across studies, while subthemes reflect more specific dimensions within these broader domains. Both the main themes and subthemes illustrate how disclosure and response are shaped by interacting interpersonal and institutional processes.

### Theme 1: relational trust, communication, and professional positioning

Theme 1 captures the relational conditions under which disclosure becomes possible and professional response becomes effective. Across 15 studies (20, 21, 23, 26–28, 31–38, 40), trust did not emerge as a singular construct but as a multidimensional process comprising relational safety, perceived competence, confidentiality, emotional validation, and continuity of care. Trust functioned both as a precondition for disclosure and as an outcome of professional conduct within the clinical encounter.

#### Subtheme 1.1: trust as relational safety and emotional validation

Women's accounts consistently positioned disclosure as contingent upon feeling safe within the clinical interaction. Safety was constructed through respectful engagement, attentive listening, and the perception that the HCP would act competently and confidentially. Studies such as Battaglia et al. (20) and Heron et al. (26) demonstrate that seemingly small interpersonal behaviours—such as sitting down, maintaining eye contact, speaking calmly, and allowing time—were interpreted as signals of attentiveness and legitimacy. These behaviours reduced perceived risk and enabled incremental disclosure.

An Italian study (28), involving 15 community midwives, indicated that most midwives recognise the importance of building a relationship with victims of DA in identifying violence. This is believed to help in creating a trusting environment that encourages victims to disclose DA. Another study (34) conducted in Australia indicated that “borrowed trust” was one of the factors influencing the disclosure of abuse. Borrowed trust illustrates how disclosure is not solely constructed within the clinical encounter but may be socially mediated through existing relational networks. It also underscores the socio-ecological interdependence between interpersonal and community-level influences shaping help-seeking behaviour.

Trust was also closely linked to validation. Validation refers to a clear acknowledgement of abuse without minimisation, disbelief, or implicit blame. Where women felt believed and supported, disclosure was facilitated. Conversely, dismissive responses, scepticism, impatience, or subtle non-verbal judgement were experienced as retraumatising and frequently resulted in withdrawal from further help-seeking (21, 23, 27, 36, 39).

Fear of not being believed, or concern that disclosure might lead to negative consequences such as child protection intervention, also inhibited openness (33, 34, 39). Validation operated at multiple levels. Emotional validation (acknowledging feelings), epistemic validation (believing women's accounts as truthful), and moral validation (clearly naming abuse as unacceptable) functioned differently within clinical encounters. Failure in any of these domains undermined relational safety. Importantly, early encounters shaped subsequent disclosure trajectories; women who experienced dismissal or disbelief were significantly less likely to re-disclose in future consultations. Importantly, first encounters were often decisive. Initial interactions shaped expectations of future engagement. Contextual factors such as the presence of a partner during consultation further complicated disclosure dynamics (34). The inability to create private space for confidential enquiry structurally inhibited disclosure, irrespective of practitioner competence. Organisational protocols that routinely ensure private consultation are therefore not procedural formalities but prerequisites for safe disclosure. When women experienced respect, privacy, and genuine concern, subsequent disclosure was more likely.

Continuity of care functioned as a relational facilitator. Longstanding relationships enabled rapport-building and gradual exploration of sensitive topics (28). However, such familiarity could also operate uncertainly; in some contexts, stigma and social proximity inhibited disclosure within established relationships (28, 31, 32, 38). Trust, therefore, was not static but negotiated within particular relational and cultural contexts.

#### Subtheme 2: professional competence, confidence, and emotional readiness

Professional positioning within a trusted role did not automatically translate into readiness to respond. Six studies (22, 30, 32, 35, 37, 38) highlighted that HCPs’ competence, confidence, and emotional readiness were distinct yet interrelated determinants of response. Competence refers to knowledge and skill, understanding indicators of abuse, safeguarding procedures, and referral pathways. Confidence reflects the perceived capability to initiate enquiry. Readiness encompasses both cognitive preparedness and emotional willingness to engage with the complexity of abuse disclosures.

Five studies (30, 32, 35, 38) illustrated that knowledge alone was insufficient. Even where training had improved theoretical understanding, practitioners described hesitation rooted in uncertainty about managing disclosure consequences. Doctors in Po-Yan Leung et al. (32) conceptualised readiness as including self-efficacy, motivational readiness, and emotional preparedness. Motivational readiness emerged as analytically distinct from competence and confidence. Even where practitioners possessed knowledge and skill, reluctance to enquire was evident when professionals anticipated limited system support or feared adverse consequences for women. Thus, the absence of enquiry cannot be interpreted solely as lack of competence; it may reflect rational appraisal of structural constraints. Similarly, district nurses in Sundborg et al. (35) described hesitation shaped by fear of causing harm, uncertainty about role boundaries, and concern about lacking appropriate follow-up resources.

Insecurity about what to do next frequently deterred enquiry (22, 36). Where HCPs felt unable to offer meaningful support beyond documentation or referral, some questioned the value of asking at all. Thus, professional competence was inseparable from system capacity. Confidence was reinforced when practitioners operated within supportive infrastructures and clear referral networks. Overall, this theme demonstrates that relational trust is co-constructed. Women assess professional demeanour, while professionals simultaneously evaluate their own preparedness and systemic backing. Disclosure and response occur at the intersection of these reciprocal assessments.

### Theme 2: educational preparedness and structural conditions of practice

Whereas Theme 1 foregrounds relational dynamics, Theme 2 captures structural determinants shaping professional readiness. Eleven studies (22, 23, 28–34, 36, 39, 40) identified educational preparation, organisational support, and system-level resources as central influences on identification and response. These factors extend beyond individual skill and situate preparedness within institutional and policy contexts.

#### Subtheme 2.1: educational preparedness and ongoing professional development

Lack of formal education regarding domestic abuse was a recurring barrier. Across 10 studies (22, 23, 28–34, 36), practitioners reported insufficient training in recognising non-physical indicators of abuse, initiating conversations sensitively, and managing disclosure outcomes. Henderson (24) highlighted that nurses often felt professionally unprepared, particularly in addressing the emotional complexity of abuse. Similarly, midwives in Mauri et al. (28) reported difficulty identifying violence unless visible injuries were present. The invisibility of psychological or coercive abuse compounded uncertainty. Training deficits extended beyond knowledge acquisition. Six studies (22, 23, 32–34, 36) emphasised that undergraduate and continuing professional education rarely addressed the affective dimension of practice—managing personal discomfort, navigating fear of escalation, or sustaining therapeutic boundaries. Consequently, even motivated practitioners expressed reluctance to initiate enquiry.

However, training was not universally ineffective. McGarry (30) and McGarry and Nairn (31) demonstrated that structured training combined with specialist support roles enhanced confidence and facilitated earlier identification. Doctors in McCauley et al. (29) expressed willingness to engage more actively if further training were available. Educational preparedness, therefore, operates on a continuum. Training that integrates knowledge, communication skills, and role clarity appears more effective than isolated informational sessions. Importantly, education must be supported by organisational structures to translate into practice.

#### Subtheme 2.2: organisational support, time, and system-level infrastructure

Preparedness was profoundly shaped by the structural realities of healthcare practice. Six studies (25, 26, 28, 33, 34, 36) identified organisational constraints, time pressures, staffing shortages, unclear reporting requirements, and limited referral pathways as significant barriers. Time emerged as both a practical and relational resource. At the consultation level, brief appointment windows limited the opportunity for sensitive enquiry. At a relational level, sustained continuity enabled trust-building over repeated encounters. Disclosure was rarely a single event; rather, it unfolded over time, contingent upon safe spaces and repeated contact.

Workload pressures constrained professionals’ capacity to explore abuse beyond presenting symptoms (29, 31). Where referral systems were unclear or community resources scarce, practitioners expressed moral distress and reluctance to initiate discussions that might not be adequately supported (22, 36). In such contexts, identification without intervention risked being perceived as ethically problematic. Importantly, the absence of overt structural barriers (such as adequate time or clear protocols) did not automatically function as a protective condition. Disclosure required the active presence of relational safety and institutional support. The absence of risk factors was not equivalent to the presence of facilitative conditions, highlighting the asymmetrical nature of barriers and enablers. Leadership and institutional culture also influenced practice. Supportive environments, clear protocols, and specialist roles (such as domestic abuse nurse specialists) were reported to legitimise enquiry and reduce professional isolation (30, 31).

Conversely, ambiguity regarding professional responsibility inhibited proactive engagement. This subtheme underscores that preparedness is not solely an individual attribute; it is structurally mediated. Without organisational commitment, protected time, and accessible referral networks, even well-trained practitioners may struggle to respond effectively.

These two themes (relational trust, communication, and professional positioning; educational preparedness and structural conditions of practice) illustrate that disclosure and response are co-constructed within relational and structural contexts. Trust, validation, and communication operate at the interpersonal level, while education, institutional culture, and system resources shape professional capacity to act.

The themes are analytically distinct but empirically interdependent. Preparedness is not reducible to training alone, nor is disclosure solely dependent on interpersonal rapport. Rather, both processes emerge from the interaction of individual readiness, relational safety, and structural support. Figure 2 visually represents the interdependent and bi-directional relationships between socio-ecological levels shaping disclosure and professional preparedness.

**Figure 2:**
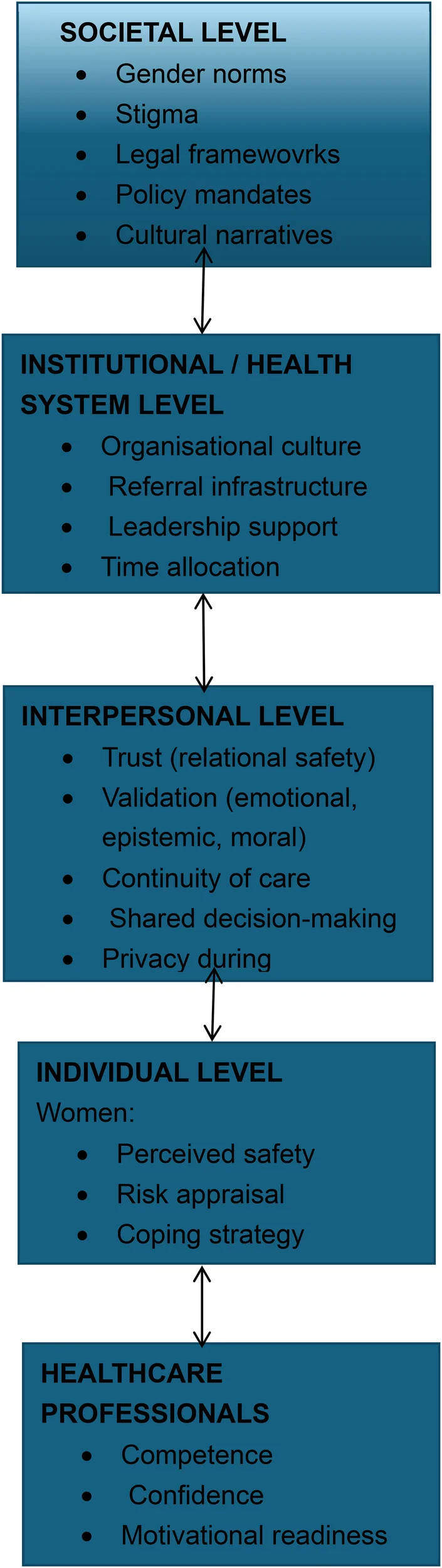
Socio-ecological model mapping of women's disclosure of DA.

## Discussion

This systematic review synthesised evidence on women's disclosure of DA within healthcare settings and healthcare professionals’ preparedness to identify and respond, interpreted through a socio-ecological framework (15). The findings indicate that disclosure and professional response are not discrete clinical events, but relational and context-dependent processes shaped by interacting influences across individual, interpersonal, institutional, and societal levels. In this respect, the review supports Gashaw et al.'s description of social systems as patterned environments of roles, relationships, and practices that structure interaction within specific settings such as healthcare services (42).

At the interpersonal level, disclosure was strongly shaped by trust, validation, and women's assessment of how healthcare professionals were likely to respond. In keeping with wider evidence, fear of not being believed and lack of trust remain central barriers to help-seeking (41). The findings of this review similarly suggest that respect for confidentiality, eye contact, shared decision-making, careful listening, and continuity of care are not incidental features of practice but central conditions through which women decide whether disclosure is safe. As previous research has shown, abused individuals often use professionals’ verbal and non-verbal responses as cues for what can and cannot be disclosed (42, 43). This means that disclosure is not simply prompted by questioning but actively shaped by the relational quality of the clinical encounter.

The findings also suggest that trust should not be understood narrowly as an individual interpersonal bond. The notion of borrowed trust usefully extends this interpretation by indicating that women may place trust in healthcare professionals through institutional legitimacy, prior social endorsement, or expectations associated with professional authority. Trust, therefore, is both relational and socially mediated. At the same time, it is fragile: Where women encounter impatience, disbelief, judgement, non-validation, or subtle emotional distancing, trust quickly deteriorates. The broader literature on victim-blaming and stigmatising narratives helps explain why disclosure may remain difficult even in health settings that are ostensibly supportive (44–46). Such narratives shape women's expectations of being misunderstood or morally judged before disclosure even occurs.

It is therefore important not to conceptualise disclosure as a straightforward or uniformly desirable endpoint. The review findings suggest that non-disclosure may, in some contexts, represent a rational and protective strategy. This interpretation is strengthened by evidence showing that women's decisions are shaped by economic dependency, housing insecurity, childcare concerns, and the broader complexity of leaving abusive relationships (47). In the UK context, disclosure may also be mediated by concerns about statutory intervention. For some women, the possibility that disclosure could trigger child protection procedures may act as a deterrent, particularly where professionals do not communicate safeguarding responsibilities clearly or sensitively (48). Moreover, leaving or separating from an abusive partner does not necessarily restore safety; rather, separation can intensify risk and produce further instability (49–51). These considerations are crucial because they demonstrate that disclosure decisions are embedded within broader material and legal realities, not simply within women's willingness to speak.

Preparedness among healthcare professionals emerged as a multidimensional construct encompassing competence, confidence, emotional readiness, and perceived role legitimacy. The findings show that knowledge alone is insufficient. Many professionals may recognise domestic abuse conceptually yet remain uncertain about what to do following disclosure, how to ask safely, or how to navigate safeguarding and referral pathways. This interpretation is consistent with previous work showing that healthcare professionals’ beliefs about domestic abuse, professional role boundaries, and emotional confidence shape the quality of response (52, 53). It also accords with qualitative meta-synthesis findings (66), which concluded that readiness is produced through the interaction of professional capability, organisational support, advocacy orientation, and system legitimacy, rather than training alone.

The distinction between competence, confidence, and readiness is important. Competence refers to knowledge and skill; confidence concerns self-perceived ability to act; and readiness includes both cognitive and emotional preparedness to engage. Existing literature indicates that deficits in training and poor role clarity weaken all three dimensions (54, 55). National and policy-oriented evidence has similarly identified inadequate education as a persistent barrier to effective domestic abuse enquiry and response (56, 57). However, the findings of this review suggest that education alone cannot resolve the problem. Practitioners may receive training and still hesitate where referral systems are unclear, institutional support is weak, or the emotional burden of asking is left unaddressed.

An important dimension absent from the included studies, but highly relevant to interpreting preparedness, is healthcare professionals’ own lived experience of domestic abuse. Dheensa et al. (67) show that healthcare professionals who are themselves survivors may have heightened sensitivity to disclosure but may also experience emotional strain, boundary difficulties, and unmet support needs in the workplace. This is significant because it suggests that preparedness is not only organisationally produced but may also be biographically shaped. In other words, readiness to respond may reflect not only what professionals know but also what they have lived through, how well supported they are, and whether workplaces recognise domestic abuse as an issue affecting staff and patients.

At the institutional level, the findings of this review clearly indicate that preparedness was structurally mediated. Time pressure, workload intensity, understaffing, and lack of continuity were repeatedly identified as conditions that constrained sensitive enquiry and limited meaningful response (58–60). Importantly, time functioned in two distinct ways: first, as a practical resource within individual consultations, and second, as relational time accumulated through continuity of care. Short consultations and fragmented care reduced opportunities to build the rapport needed for safe disclosure, whereas continuity enabled trust-building and more nuanced understanding over time. These findings reinforce the argument that disclosure often unfolds gradually, not as a one-off event, and that system design materially affects whether this process can occur.

This institutional interpretation is further strengthened by wider literature demonstrating that structural barriers within healthcare systems can inhibit identification and follow-up even where professionals are motivated to respond (58, 60). Post-pandemic service pressures and workforce strain have further intensified such constraints within the NHS, reducing the availability of protected time and increasing the likelihood that domestic abuse enquiry will be deprioritised in practice (61, 62). Under these conditions, it is not enough to exhort professionals to ask; healthcare systems must actively enable them to do so.

The findings also show that preparedness depends on whether domestic abuse work is recognised as a legitimate and supported component of healthcare practice. Lack of referral clarity, unclear reporting requirements, weak interagency links, and uncertainty about responsibilities undermine action even where concern is present (63). Conversely, evidence from structured intervention programmes demonstrates that when training is combined with system-level support, referral infrastructure, and organisational endorsement, identification and referral can improve (64). This is an important distinction: The challenge is not only educational but also organisational and political.

At the societal level, gender norms, stigma, and institutional narratives continue to shape both disclosure and response. Domestic abuse is often still positioned as a private or marginal issue despite its clear health consequences. This can lead to what might be called institutional minimisation, where domestic abuse is acknowledged rhetorically but not prioritised practically. The socio-ecological model is especially useful here because it highlights how interpersonal failures are often symptoms of wider structural misalignment rather than simply individual professional shortcomings (15). The findings of this review therefore support an ecological understanding of preparedness: It is not a static personal trait, but an effect of relationships, organisational conditions, legal frameworks, and professional cultures operating together.

Overall, this review suggests that improving responses to domestic abuse requires more than increasing awareness or delivering isolated training sessions. Sustainable improvement depends on alignment across socio-ecological levels: relationally safe interactions, professionals who are competent and emotionally supported, organisational systems that provide time and referral capacity, and broader service cultures that recognise domestic abuse as a legitimate healthcare concern (52, 53, 63–65). Without such alignment, efforts to encourage disclosure risk placing responsibility on individual women and individual practitioners while leaving the structural conditions of silence intact.

## Strengths and limitations

This review has several methodological strengths. It was conducted and reported in accordance with PRISMA guidance (11) and employed a structured search strategy informed by the SPIDER framework, which is well suited to qualitative evidence synthesis. The use of SPIDER ensured alignment between the review's focus on lived experience, healthcare professionals’ preparedness, and qualitative research designs. Clear inclusion and exclusion criteria were applied, and screening and data extraction processes incorporated independent verification, enhancing transparency and reducing the likelihood of systematic error.

Methodological quality was appraised using the CASP tool (12), and quality considerations were incorporated into the interpretive process rather than used as exclusion criteria. This approach is consistent with qualitative systematic review methodology, allowing conceptually relevant studies to be retained while maintaining critical awareness of variation in methodological rigour. The use of the socio-ecological model (15) as an interpretive framework represents a further strength, enabling integration of findings across individual, interpersonal, institutional, and societal levels. This facilitated a more analytically coherent understanding of how disclosure and professional response are shaped by interacting systems rather than isolated factors.

Several limitations should be considered. First, although the search strategy was comprehensive, it was not sufficiently sensitive to capture studies examining healthcare professionals’ own lived experiences of domestic abuse. This is an important omission, as emerging evidence indicates that such experiences may shape empathic engagement, emotional burden, and response behaviours in clinical practice. The absence of this dimension limits the extent to which preparedness can be understood as a biographically and relationally situated construct and highlights an important area for future research.

Second, the review included only English-language publications and excluded grey literature, which may have introduced publication and language bias. Most included studies were conducted in high-income settings, limiting transferability to contexts with different healthcare infrastructures, legal frameworks, and safeguarding systems. Variations in reporting requirements, referral pathways, and cultural norms may shape both disclosure and professional response in ways not fully captured in this synthesis.

Third, while steps were taken to enhance methodological rigour, including partial double-screening and independent verification of data extraction, not all studies were screened in duplicate. Although this approach is recognised within qualitative systematic reviews, it may introduce a degree of selection bias. However, consensus processes and cross-checking procedures were used to mitigate this risk and enhance the dependability of the findings.

Finally, while the review conceptualises disclosure as relational and socially mediated, it does not examine longitudinal trajectories of disclosure or systematically analyse coping strategies over time. Future research should explore how disclosure decisions evolve across the life course and how healthcare interactions intersect with broader social and structural dynamics.

## Conclusion

This systematic review demonstrates that women's disclosure of domestic abuse within healthcare settings is not a discrete or purely individual act, but a relational and context-dependent process shaped by trust, perceived safety, and expectations of professional response. Disclosure emerges through the interaction of interpersonal dynamics, professional preparedness, organisational conditions, and wider social and legal contexts. Healthcare professionals’ readiness to identify and respond to domestic abuse is similarly multifaceted. It is not reducible to knowledge or training alone but reflects the interaction between competence, confidence, emotional readiness, and the availability of organisational and system-level support. In the absence of clear referral pathways, protected time, and institutional backing, even well-informed practitioners may be constrained in their ability to respond effectively.

The findings underscore that improving responses to domestic abuse requires coordinated action across socio-ecological levels. Relationally, healthcare encounters must prioritise trust, validation, confidentiality, and shared decision-making. Organisationally, services must provide the structural conditions necessary for safe enquiry, including continuity of care, clear protocols, and accessible referral systems. At the system level, leadership, policy alignment, and interagency collaboration are essential to support consistent and effective responses. Importantly, disclosure alone should not be assumed to result in improved outcomes. Without adequate system readiness, disclosure may expose women to additional risks, including procedural harm, unmet expectations, or unintended consequences associated with safeguarding processes. Efforts to promote disclosure must therefore be accompanied by parallel investment in system capacity and trauma-informed care.

Future research should extend this work by examining healthcare professionals’ own experiences of domestic abuse and how this shape clinical practice, as well as by exploring disclosure processes in diverse healthcare and socio-cultural contexts. A more comprehensive understanding of preparedness must account not only for professional training and organisational systems but also for the relational and biographical factors that influence how care is delivered. Overall, meaningful improvement in healthcare responses to domestic abuse will depend on moving beyond individual-level interventions towards integrated, system-wide approaches that align relational practice with organisational and structural support.

## Data Availability

The original contributions presented in the study are included in the article/Supplementary Material; further inquiries can be directed to the corresponding author.
